# Live functional assays reveal longitudinal maturation of transepithelial transport in kidney organoids

**DOI:** 10.3389/fcell.2022.978888

**Published:** 2022-08-15

**Authors:** Astia Rizki-Safitri, Navin Gupta, Ken Hiratsuka, Kenichi Kobayashi, Chengcheng Zhang, Kazumi Ida, Lisa M. Satlin, Ryuji Morizane

**Affiliations:** ^1^ Nephrology Division, Massachusetts General Hospital, Boston, MA, United States; ^2^ Department of Medicine, Harvard Medical School, Boston, MA, United States; ^3^ Wyss Institute for Biologically Inspired Engineering, Harvard University, Cambridge, MA, United States; ^4^ Department of Pediatrics, Icahn School of Medicine at Mount Sinai, New York City, NY, United States; ^5^ Harvard Stem Cell Institute, Cambridge, MA, United States

**Keywords:** real-time imaging, functional assay, tubular transport, organoid, kidney, nephron

## Abstract

Kidney organoids derived from hPSCs have opened new opportunities to develop kidney models for preclinical studies and immunocompatible kidney tissues for regeneration. Organoids resemble native nephrons that consist of filtration units and tubules, yet little is known about the functional capacity of these organoid structures. Transcriptomic analyses provide insight into maturation and transporter activities that represent kidney functions. However, functional assays in organoids are necessary to demonstrate the activity of these transport proteins in live tissues. The three-dimensional (3D) architecture adds complexity to real-time assays in kidney organoids. Here, we develop a functional assay using live imaging to assess transepithelial transport of rhodamine 123 (Rh123), a fluorescent substrate of P-glycoprotein (P-gp), in organoids affixed to coverslip culture plates for accurate real-time observation. The identity of organoid structures was probed using Lotus Tetragonolobus Lectin (LTL), which binds to glycoproteins present on the surface of proximal tubules. Within 20 min of the addition of Rh123 to culture media, Rh123 accumulated in the tubular lumen of organoids. Basolateral-to-apical accumulation of the dye/marker was reduced by pharmacologic inhibition of MDR1 or OCT2, and OCT2 inhibition reduced the Rh123 uptake. The magnitude of Rh123 transport was maturation-dependent, consistent with MDR1 expression levels assessed by RNA-seq and immunohistochemistry. Specifically, organoids on day 21 exhibit less accumulation of Rh123 in the lumen unlike later-stage organoids from day 30 of differentiation. Our work establishes a live functional assessment in 3D kidney organoids, enabling the functional phenotyping of organoids in health and disease.

## Introduction

Kidney organoids derived from human pluripotent stem cells (hPSCs) provide three-dimensional (3D) tissue models for disease modeling, also serving as a potential future source for kidney transplantation. Immunolocalization studies demonstrate filtration and reabsorptive units in kidney organoids that morphologically resemble segmented nephrons of the native kidney and tubular segments therein express transport proteins known to mediate transepithelial transport (reabsorption and secretion) in the fully differentiated mammalian kidney (Taguchi et al., 2014; Freedman et al., 2015; Morizane et al., 2015; Takasato et al., 2015; Wu et al., 2018; Homan et al., 2019; Subramanian et al., 2019; Miyoshi et al., 2020). While multiple studies have demonstrated the utility of kidney organoids to study a variety of kidney diseases, single-cell and bulk transcriptomic analyses suggest a relative immaturity of the organoids with gene expression profiles similar to those of 1st ∼ 2nd trimester fetal kidneys (Garreta et al., 2019). Critical to future studies in organoids is the need to demonstrate and validate physiological function in kidney organoids.

The (Re)Building a Kidney (RBK) consortium led by the National Institute of Diabetes and Digestive and Kidney Diseases (NIDDK) hosts ∼20 institutions across the United States and is focused on a wide range of projects related to kidney regeneration and repair. One RBK priority is to develop a data repository for functional assessments that would be accessible to researchers worldwide who wish to functionally phenotype their kidney models including kidney organoids (ReBuilding a Kidney). Functional assessments will determine whether kidney cells and tissues are appropriate for modeling disease and future transplantation. Assays proposed to be included in this repository cover those aimed at measuring glomerular (creatinine clearance, dextran filtration) and tubular (e.g., absorption of water, sodium, glucose, and dextran-albumin; secretion of organic ions) functions (Rizki-Safitri et al., 2021) in kidney models. Some established assays can be modified to cultured cells, yet it is challenging to apply these functional assessments to complex 3D tissues such as kidney organoids.

Kidney organoids possess a multicellular configuration and 3D architectures which limit the imaging capability in real-time. The requirement to generate reporter lines for real-time imaging presents an additional hurdle to perform functional assays in kidney organoids. Here, we develop a simple live imaging setup for monitoring of basolateral-to-apical transepithelial transport of Rhodamine123 (Rh123) in kidney organoids labeled with a proximal tubule-specific lectin. Using Rh123, an organic compound, our live imaging studies show vectorial transport of Rh123 taken up at the basolateral membrane by organic cation transporters (OCTs) and secreted into the tubular lumen by the apical export protein multidrug resistance protein 1 (MDR1) in kidney organoids and that this transport process is developmentally regulated.

## Results

### Live imaging set up for 3D kidney organoids

**FIGURE 1:**
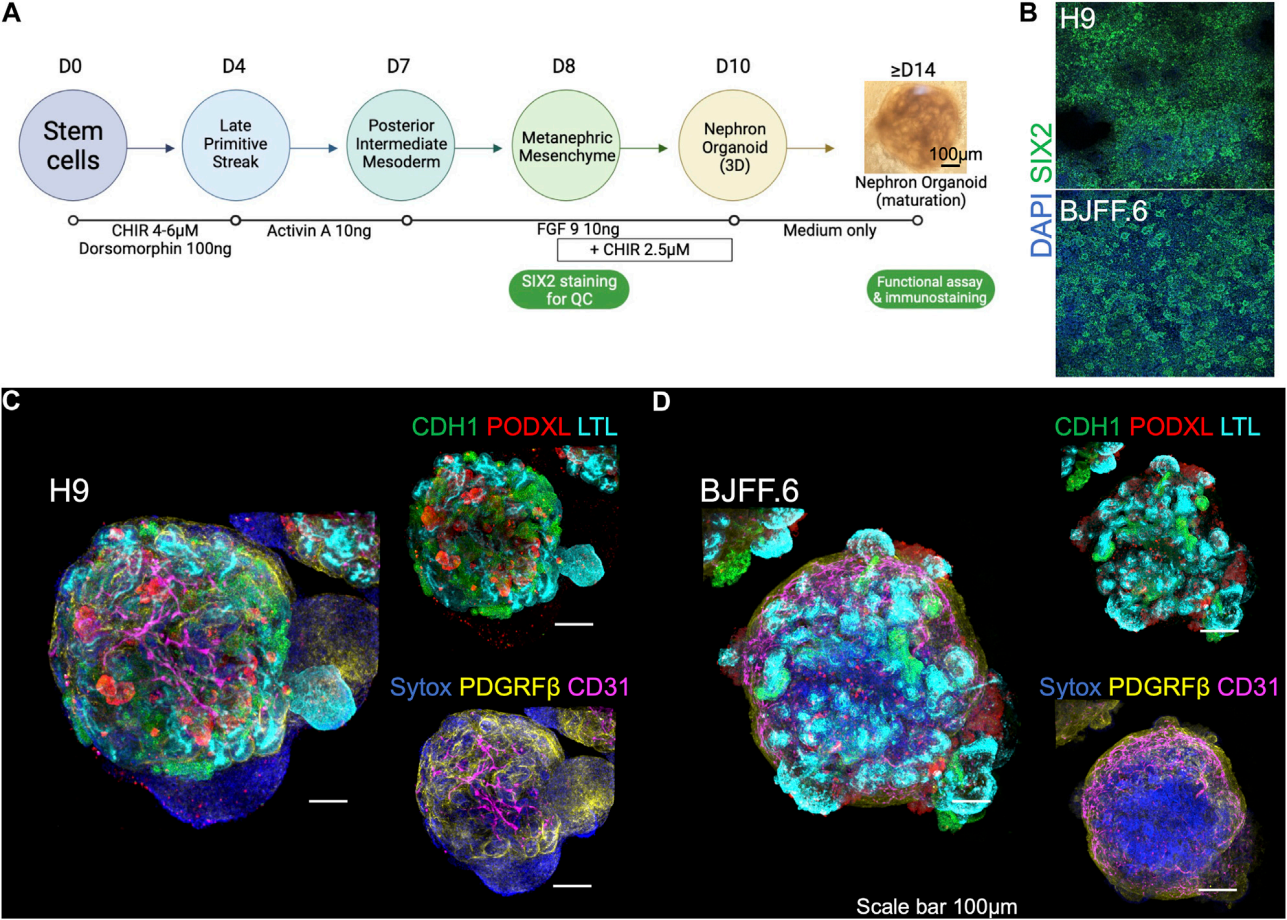
Kidney organoids utilized for live imaging functional assay. **(A)** Steps in generating kidney organoids using the 6-step differentiation protocol (Morizane et al., 2015; Morizane and Bonventre, 2017). Organoids at > d14 are used for live imaging functional assays. **(B)** SIX2 staining is used to confirm the quality of kidney organoid differentiation on embryonic (H9) and induced (BJFF.6) stem cell lines. **(C)** The d21 kidney organoids derived from both lines form a segmentation of nephrons as presented by CDH1^+^, PODXL^+^, and LTL^+^ cells, as well as containing **(D)** PDGRFβ^+^ stromal and CD31^+^ endothelial cells. FGF9: fibroblast growth factor 9. LTL, lotus tetragonolobus lectin; PODXL, podocalyxin; CDH1, cadherin-1; PDGFRβ, platelet-derived growth factor receptor beta.

H9 (female human embryonic), and BJFF.6 (male human induced pluripotent) stem cells were differentiated into kidney organoids using a previously established protocol with minor modifications (Morizane et al., 2015; Morizane and Bonventre, 2017). First, SIX2^+^ nephron progenitor cells (NPCs) were induced by sequential treatment with CHIR for 4 days, Activin for 3 days, and FGF9 for 1 day (Figure 1A). All batches of differentiation experiments were quality-controlled at this NPC stage by immunocytochemistry for SIX2 (Figure 1B). NPCs were then dissociated and resuspended in 96-well U-bottom plates in the differentiation medium supplemented with CHIR and FGF9. After 2 days of incubation, CHIR was removed from the medium, and organoids were cultured until day 14 of differentiation with FGF9. After that, kidney organoids were spontaneously differentiated in the basic medium alone and harvested for immunofluorescence microscopy analyses. Organoids established from H9 (Figure 1C) and BJFF.6 (Figure 1D) contained multiple segmented nephron structures that resemble glomeruli and tubules marked by PODXL and LTL/CDH1 respectively. Additional immunostaining for stromal cells identified PDGFRβ^+^ interstitial fibroblasts/pericytes and CD31^+^ endothelial cells in the interstitium of kidney organoids. 3D images reconstructed from z-stacks with tissue clearing showed lumen formation in tubular segments (Supplementary Videos S1A,B).

**FIGURE 2:**
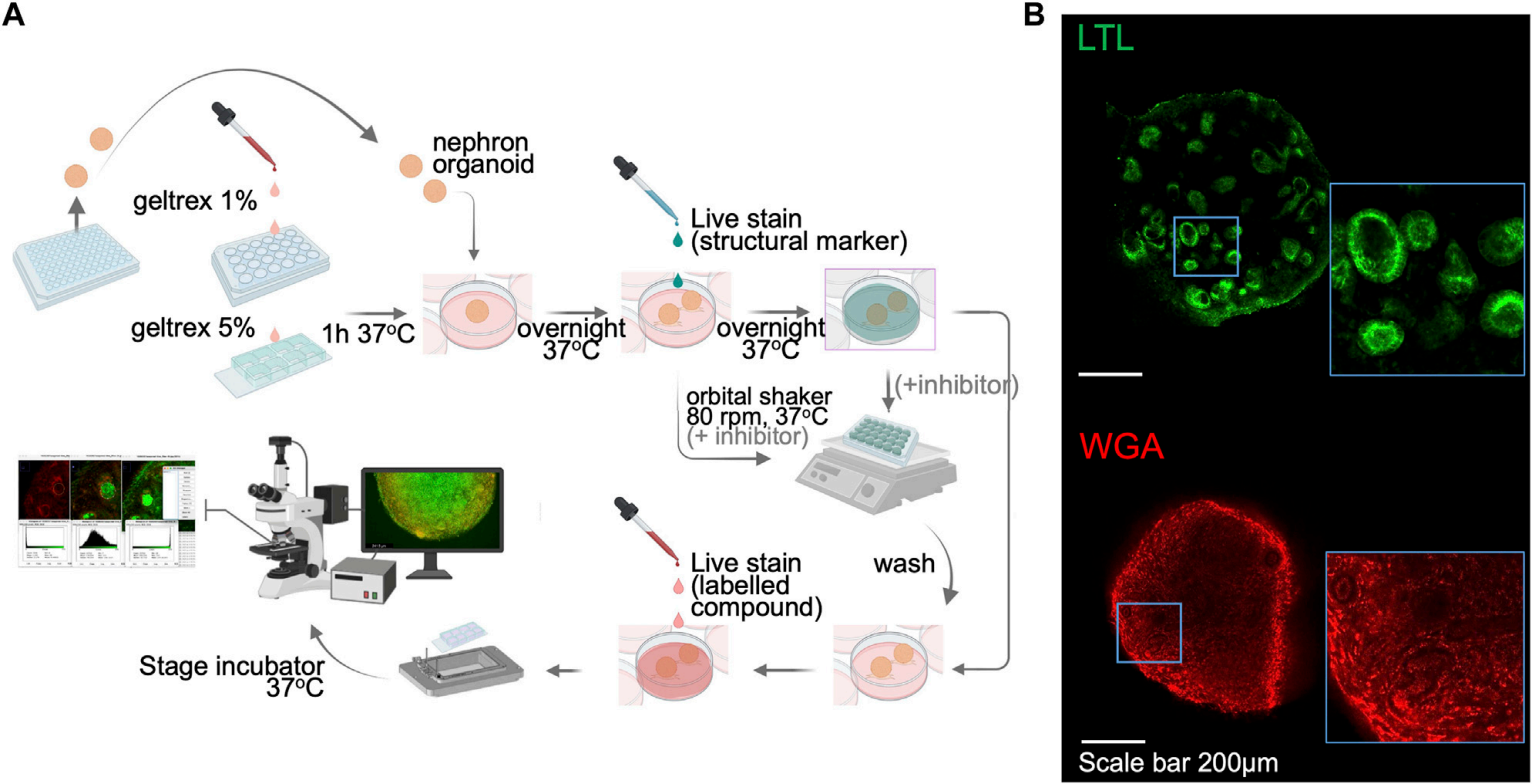
Sample preparation and setup for kidney organoid functional assays by live imaging. **(A)** A schematic illustration of the live imaging protocol. Following the organoid attachment on culture plate/chamber slides, structural markers were utilized to help distinguish nephron segmentation. The orbital shaker allows the live staining dyes to penetrate into the kidney organoid. The stage-top incubator maintains the culture environment of live organoids and enables real-time functional assay. **(B)** Immunofluorescent images of live LTL and WGA staining in kidney organoids. WGA, wheat germ agglutinin.

One limitation in organoid research is the difficulty of live imaging due to the tissue thickness. Each kidney organoid is comprised of approximately 100,000 cells, forming a spheroid that measures ∼1 mm in diameter in suspension culture. For thick tissues, multi-photon imaging systems are necessary to visualize the internal structures, yet such systems are not readily available in many research institutions. In addition, during the suspension culture, kidney organoids move and rotate, making it impossible to capture sequential images of the same nephron structures in real-time. Here, we optimize a live imaging setup that enables the functional evaluation of kidney organoids. Kidney organoids transferred onto Geltrex-coated chamber slides from 96-well low adhesion plates adhere to the coated surface by the next day (Figure 2A). This step helps to fix the organoid in a given position and allows functional assessment of the same nephron structures of interest over time. After the adhesion, organoids are slightly flattened, yet their 3D spheroid architecture is still preserved. We then applied a variety of live imaging dyes specific for nuclei and cell membranes, and lotus tetragonolobus lectin (LTL) and wheat germ agglutinin (WGA) conjugated with fluorescent tags (Figure 2B) to enable the identification of tubular structures in live organoids. A nuclear dye was also added to the bath medium to label nuclei in organoids, yet we notice weaker signals in tubular cells than in surrounding stromal cells. Immunocytochemistry after 4% paraformaldehyde (PFA) fixation confirmed segmented nephron structures, similar to organoids cultured in suspension, with stromal cells adhering to the chamber slide (Supplementary Figure S1). Importantly, the single-photon confocal is capable of acquiring live images up to 100 μm in depth, which is enough to capture tubules that are typically under 50 μm in diameter. This live imaging setup allows researchers to assess tubular transport activities in kidney organoids in real-time without the need for reporter lines.

### Live organoid imaging visualizes the tubular secretion activity

Within the fully differentiated kidney, proximal and distal tubules maintain homeostasis by secretion of wastes and drugs, and reabsorption of essential nutrients (Curthoys and Moe, 2014; Subramanya and Ellison, 2014). Proximal tubules themselves are responsible for reabsorbing ∼60–70% of the glomerular filtrate. Among many of the processes, proximal tubules mediate transepithelial absorption of Na, HCO_3_^−^, glucose, albumin, and many more solutes (Curthoys and Moe, 2014). Proximal tubules also play a crucial role in the secretion and thus elimination of drugs and toxins (Wang and Kestenbaum, 2018). MDR1 is an apical p-glycoprotein efflux transporter that mediates drug secretion into the lumen of the proximal tubule (Chin et al., 1989). The activity of this transport protein has been visualized by monitoring the transport of rhodamine 123 (Rh123), a fluorescent MDR1 substrate. Rh123-inhibitor assays are standard to evaluate MDR1 activity (Solvo Biotechnology) and have been successfully applied to proximal tubular cell lines (Wen et al., 2014) and tubuloid-derived cells (Schutgens et al., 2019) as well as in liver duct cells (Rizki-Safitri et al., 2020).

**FIGURE 3:**
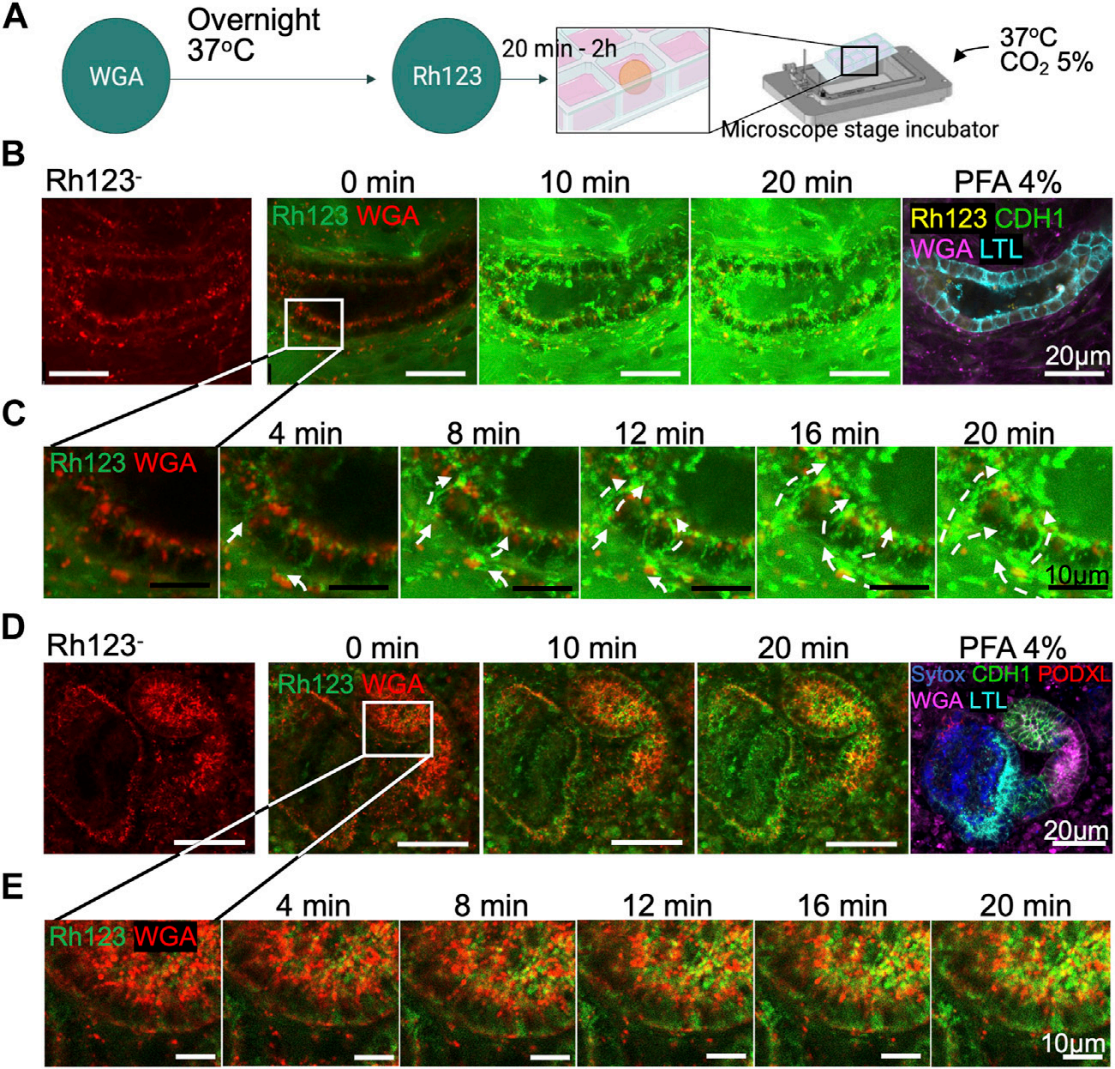
Transepithelial transport in live kidney organoids. **(A)** Protocol for Rh123 assay using the microscope stage incubator. **(B)** Rh123 transport into tubular lumen after 20 min incubation. The tubular structure was later confirmed as an LTL^+^ proximal tubule after organoid PFA fixation. **(C)** High magnification images display a distinct Rh123 trans-cellular transport from the interstitial to luminal space of proximal tubules. The dashed lines show the Rh123 movement, and the arrowhead indicates the transport direction. **(D**,**E)** In contrast, tubular structures that transport less Rh123 are CDH1^+^ distal tubules as confirmed by immunostaining.

After adhesion of day 30 < organoids to chamber slides and incubation with WGA lectin, we set up live imaging in a stage-top incubator that maintains the humidity, the temperature at 37°C and 5% CO_2_ environment (Figure 3A). Tubular structures were first identified by WGA live imaging in the absence of Rh123 (Rh123^−^), and x-y-z positions were set and programmed to acquire images of the same regions of organoids over time. Then, Rh123 was added (Rh123^+^) to the culture medium (time point = 0 min). Live images were acquired every 2 min until the Rh123 signal was detected in the tubular lumen. After the addition of the dye to the culture medium, Rh123 signals quickly increased in the interstitial space of kidney organoids. Rh123 signals were then detected in the tubular cytoplasm, followed by an increase in the signal in the tubular lumen by 20 min of incubation (Figure 3B, Supplementary Video S2). Higher magnification images demonstrated the transepithelial transport of Rh123 from the basal side to the lumen (Figure 3C), consistent with what is known about vectorial transport of this substrate in native kidneys. Once the transport assay was complete, the samples were fixated and stained for LTL, CDH1, and PODXL to confirm the segment identity (Figure 3B right). While LTL^+^ tubules showed active transepithelial transport of Rh123 (Figure 3C), we did not observe such activity in CDH1^+^ tubules (Figures 3D,E). This observation suggests that proximal tubules but not distal tubules are responsible for the secretion of drugs and organic compounds, as has been described in kidneys *in vivo* (Motohashi et al., 2002; Breljak et al., 2016).

### Multidrug resistance protein 1 and organic cation transporters 2 mediate rhodamine 123 secretion in organoid tubules

**FIGURE 4:**
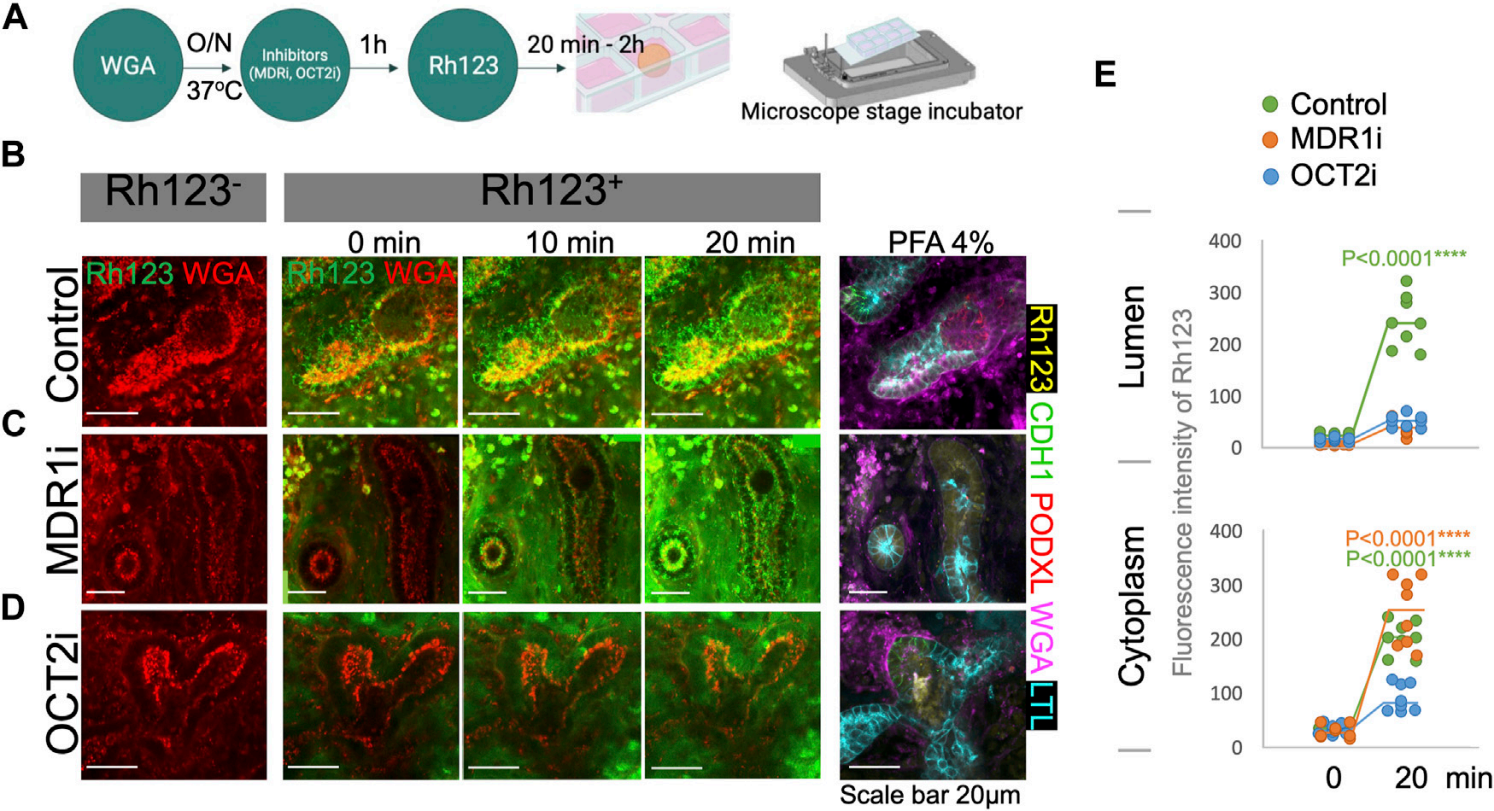
Rh123 transport is mediated by MDR1 and OCT2 in kidney organoids. **(A)** Protocol for Rh123 assay using transporter inhibitors. **(B)** Rh123 transport into the lumen of proximal tubules in the control samples. **(C)** MDR1i blocks luminal secretion of Rh123, resulting in fluorescence accumulation in the apical cytoplasmic region. **(D)** OCT2i decreases the Rh123 luminal secretion and cellular uptake. **(E)** Quantification of the Rh123 intensity in the tubular lumens and cytoplasm *n* = 6. ****: *p* < 0.0001.

To validate whether MDR1 is responsible for Rh123 efflux from the cytoplasm into the lumen, we tested the effect of an MDR1 inhibitor (MDR1i) PSC-833 (valspodar) (Wen et al., 2014; Schutgens et al., 2019). Kidney organoids were pretreated with MDR1i for 1 h before the live imaging (Figure 4A). Rh123 signals were detected in the tubular cytoplasm of MDRi-treated organoids after 2 min of incubation; however, luminal Rh123 signals were significantly reduced by MDR1i when compared to controls after 20 min (Figures 4B,C; Supplementary Videos S3A,B). In MDR1i-treated samples, Rh123 accumulated in the subapical region of the cells, suggesting basolateral uptake and intracellular movement to the apical side of the cell, indicated by white arrows (Figure 3C), were not affected by MDR1i. We later confirmed that the efflux of Rh123 into the lumen was decreased by MDR1i, primarily in the LTL^+^ proximal tubules after fixation and immunostaining for nephron markers (Figures 4B,C right).

While our assay shows transepithelial transport in the proximal tubules of kidney organoids, little is known about the mechanism of basolateral uptake of Rh123 in kidney tubules. OCT2 plays an important role in organic cation uptake from the interstitium in proximal tubules (Wang and Kestenbaum, 2018; Koepsell, 2020). Rh123 is a lipophilic cationic compound that is likely to be transported by OCT2. Indeed, one study that overexpressed hOCT2 in HEK293 cells showed enhanced Rh123 uptake into the cytoplasm (Jouan et al., 2014). To investigate the uptake mechanism in human kidney organoids, an OCT2 inhibitor (OCT2i), Tetrapentylammonium (TPA), was examined in organoid tubules (Jansen et al., 2019). The OCT2i-treated organoids showed substantially reduced signals of Rh123 in both the tubular lumen and cytoplasm of proximal tubular structures (Figure 4D, Supplementary Video S3C).

To determine the statistical significance of the Rh123 transport in the presence or absence of MDR1i or OCT2i, we quantified the fluorescence intensities from real-time observation in regions of interest (ROI) marked in the tubular lumens or cytoplasm. We compared the Rh123 signals between 0 and 20 min incubations, and the values were normalized against the Rh123^−^. Quantitation of fluorescence intensity confirms a significant accumulation of Rh123 in the lumens of control kidney organoids after 20 min incubation (Figure 4E top). Exposure of organoids to MDR1i enhanced the accumulation of Rh123 in the cytoplasm, predominantly in the subapical region (Figure 4E bottom), whereas the Rh123 intensities in the cytoplasm were significantly reduced by OCT2i treatment. These simple quantitative assessments enable statistical evaluation and are consistent with basolateral uptake of Rh123 via OCT2 and luminal efflux mediated by MDR1 in live kidney organoids.

### Live functional assay elucidates functional maturation of kidney organoids

**FIGURE 5:**
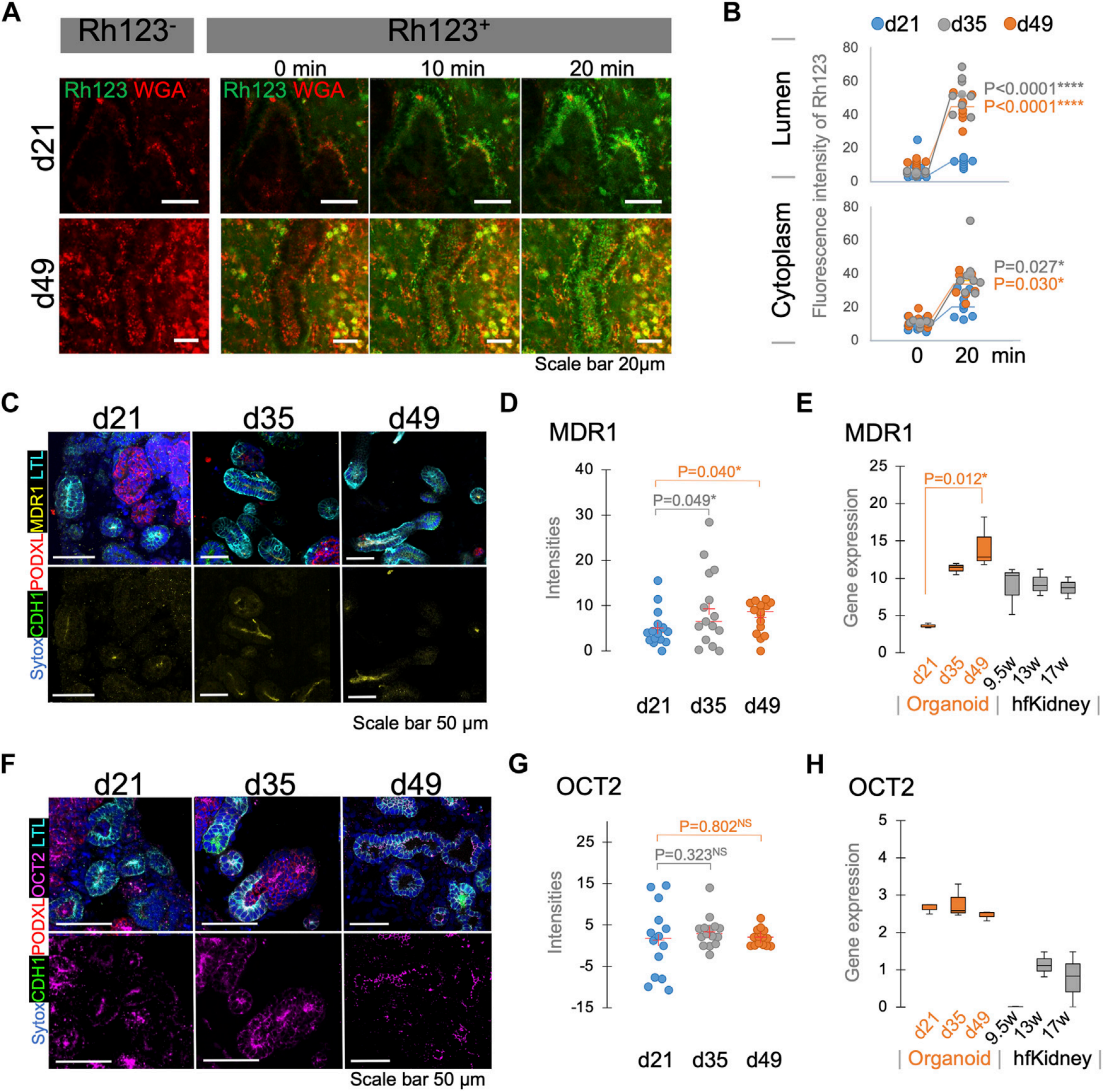
Functional maturation assessed by live functional assay, immunostaining, and RNA-seq in kidney organoids. **(A)** Rh123 transport in proximal tubules of kidney organoids between d21 and d49. Organoids at d49 show prominent luminal accumulation which is later **(B)** confirmed by quantification between 0 and 20 min time points. **(C)** Immunostaining validates that MDR1 expression on d35/49 is greater than on the d21. MDR1 expression is localized to the apical membrane of the proximal tubules. **(D)** Quantification of MDR1 staining confirms a significant increase in MDR1 expression on d35/49 *n* = 15. **(E)** RNA-seq for MDR1 in kidney organoids and human fetal kidneys (hfKidney). **(F)** Immunostaining for OCT2 in d21, d35, and d49 kidney organoids. **(G)** Quantification of OCT2 staining shows stable expression from d21. *n* = 15. **(H)** RNA-seq for OCT2 in kidney organoids and hfKidney. *n* = 3 for organoid samples and 2 for hfKidneys.

Bulk RNA-seq analysis in our recent study suggests that kidney organoids mature during 7 weeks of differentiation in culture (Gupta et al., 2022). To evaluate the functional maturation of tubular transport, we compared the Rh123 transport activities in kidney organoids longitudinally during this period of differentiation, and specifically on days 21 (d21), 35 (d35), and 49 (d49). Basolateral uptake of Rh123 was detected at all stages of differentiation (Figure 3B, Figure 5A, Supplementary Videos S4A,B); however, the luminal accumulation of Rh123 after 20 min was significantly higher in d35-49 organoids than in d21 (Figure 5B top). The Rh123 signal in the tubular cytoplasm of d35-49 organoids was also significantly higher than d21 (Figure 5B bottom). These results are consistent with functional maturation of this organic cation transport pathway in organoids cultured over time, with more mature proximal tubules demonstrating an increased ability to transport Rh123.

To confirm that the expression of the relevant transport proteins is developmentally regulated, we assessed MDR1 and OCT2 expression by immunostaining and compared them with bulk RNA-seq data of d21, d35, and d49 organoids. Notably, the MDR1 expression was higher on d35-49 than on d21, and its localization was specific to the subapical region of LTL^+^ proximal tubular cells (Figures 5C,D). RNA-seq showed lower expression on day 21 than later time points at day 35–49 (Figure 5E). These results were consistent with a developmental regulation of expression of MDR1 at the level of message in proximal tubules.

In contrast, OCT2 was expressed at all stages of differentiation (Figure 5F) and the intensity of staining did not show a significant increase during organoid maturation (Figure 5G). RNA expression also showed the same levels of OCT2 expression in all stages of differentiation (Figure 5H). These results suggested that OCT2 is not solely responsible for Rh123 uptake in proximal tubules. Indeed, OCT2i did not completely block the Rh123 uptake in LTL^+^ tubules (Figures 4D,E). Human fetal kidney samples showed the expression of both MDR1 and OCT2 from 1st trimester, consistent with those expressions in kidney organoids (Figures 5E,H) (Han et al., 2018).

## Discussion and outlook

Real-time assessment of physiological function of kidney organoids is challenging due to their structure, thickness, and tissue complexity. Ideally, functional assays should be performed in a given organoid structure over time (before and then after the addition of a drug, during maturation). This requires the investigator to be able to interrogate/analyze the same segment and cells over time, a challenging task especially when assays require manipulations (e.g., mixing on an orbital shaker) off the microscope. Here, we use real-time imaging to perform a functional assessment of transepithelial transport over time in proximal tubules in kidney organoids “immobilized” on a plate/chamber coated with Geltrex. The data obtained include real-time fluorescence changes that can be analyzed in both qualitative and quantitative manners.

In this study, we validate that kidney organoids are capable of basolateral-to-luminal secretion as evidenced by the luminal accumulation of Rh123, initially added only to the bath solution, vectorial transport which can be blunted by treatment with transporter inhibitors. The observation that the tubular segments exhibiting Rh123 secretion were LTL^+^ suggests that the transepithelial transport occurred predominantly in proximal tubules and is less likely in distal tubules/other cells. This is consistent with the site of organic ion transport in the native kidney. Specifically, in the fully differentiated mammalian kidney, the proximal tubules express various transporters that can transport drugs (Nigam, 2015).

Our results showed that MDR1 and OCT2, responsible for eliminating drugs and xenobiotics in the mammalian kidney (Wang and Kestenbaum, 2018), were functional in kidney organoids after day 21 in culture. Furthermore, we showed that the molecular and functional expression of MDR1 and OCT2 are developmentally regulated in organoids. Apical expression of MDR1, abundant in differentiated organoids, increased with advancing days in culture. The observation that OCT2i treatment reduced, but did not eliminate, luminal and cytoplasmic accumulation of Rh123 suggests that basolateral Rh123 uptake is partially mediated by OCT2. Residual Rh123 uptake in the presence of OCTi inhibitors suggests the presence of alternate basolateral uptake pathways for this molecule. Nonetheless, our results show that organoids express OCT2, consistent with previous reports that organoids were able to recapitulate kidney injury induced by cisplatin, a substrate of OCT2 (Morizane et al., 2015; Digby et al., 2020; Gupta et al., 2022).

Examination of data from kidney transcriptomic analyses (KIT) (Humphreys) and the kidney precision medicine project (KPMP) (KPMP) suggest that other transporters that might mediate basolateral transport are OCT1, OCT3, and possibly anion transporters such as OAT1. However, these transporters appear to be expressed at early stages of organoid maturation. As the appearance of message, protein and then function for a transporter may not be concurrent, future studies may focus on examing the development and regulation of functional activity of these transport proteins.

One limitation of this study is that our method relies on the fluorescence signal of the live cell staining, thus not applicable to unconjugated markers. The live staining imaging tends to have the background noise which may be misinterpreted as uptake of the dye by interstitial cells. Additionally, not every structure has a live cell stain to act as a marker, for example, podocytes, limiting this assay only to tubules at this time. Another limitation of this method is that it cannot be applied to assess kidney function of luminal reabsorptive transport pathways, such as that mediating dextran and albumin uptake because access to the tubular lumen is limited. To this end, techniques such as microperfusion of isolated tubules or studies in microdissected and split-open tubules (with access to the apical membrane) (Satlin and Palmer, 1997; Carrisoza-Gaytan et al., 2020; Rein et al., 2020) would be necessary to assess dextran or albumin reabsorption from the tubular lumen.

Collectively, our study suggests that kidney organoids are functional and can mediate transepithelial transport. To determine the degree of functionality, further studies are necessary using human adult and embryonic kidneys as controls. While our study focused on transepithelial transport of Rh123, mediated by a proximal tubule organic cation secretory pathway, we propose that our method may be useful in guiding future functional phenotyping of additional structures in kidney organoids and kidney models and assessing the quality of bioengineered kidneys generated by various protocols. It could also be relevant in assessing function in the 3D-tissue model of other organs which express the epithelial transporters, such as in the intestine, bile duct, and blood vessel organoids.

## Materials and methods

### Pluripotent stem cells and kidney organoid culture

H9 human embryonic stem cells (ESCs; WiCell) and BJFF.6 human induced PSCs (provided by Prof. Sanjay Jain at Washington University) were maintained in StemFit Basic02 (Ajinomoto Co., Inc.) supplemented with 10 ng/ml FGF2 (Peprotech) as previously described (Morizane et al., 2015; Morizane and Bonventre, 2017). Stem cells at passages 34–55 were used for the experiment. Kidney organoids were generated using a previously reported protocol (Morizane et al., 2015; Morizane and Bonventre, 2017). Briefly, hPSCs were differentiated into SIX2^+^ nephron progenitor cells (NPCs) in 2D culture with 80–90% efficiency. The NPCs were later transferred to 3D cultures in 96-well ultra-low attachment plates (Corning) and differentiated into nephron organoids. Organoids at d21-49 were used for functional assays.

### Sample preparation on the plate/chamber slides

24-well plates (Corning) and chamber slides (Ibidi) were used for live imaging. 1 and 5% Geltrex (Gibco) coating was done on 24-well plates and chamber slides respectively, with a 1-h incubation at 37°C. After removal of the Geltrex coating solution, organoids were placed on the bottom of the wells or chamber slides and incubated at 37°C overnight. It is important to keep the medium at low levels to prevent organoids from floating off the bottom. The attachment of organoids to the glass bottom was confirmed using a phase-contrast microscope with morphological assessment on stromal-like cell sprouting and spreading on the surface of the well/chamber slides.

### Live imaging set up

Plates/chamber slides containing live stained kidney organoids were placed into an STX-WSKMX series stage incubator system (Tokai Hit) following the manufacturer’s manual. Samples were incubated at 37°C with 5% CO2 in the STX’s chamber that was installed on the confocal microscope stage (Leica Stellaris-8) for real-time imaging.

### Transepithelial transport assay on proximal tubules

The attached organoids were incubated overnight at 37°C with 5 μg/ml of WGA conjugated-647 (biotium) added to the bath in an orbital shaker at 80 rpm. LTL can be used as a marker to specifically visualize proximal tubules. As indicated, organoids were incubated for 1 h with the inhibitors 20 μg/ml PSC-833 (Sigma) or 1 mM Tetrapentylammonium chloride/TPA (Sigma). Rhodamine 123 (Rh123, Sigma) was added to the live imaging medium at a concentration of 10 µM immediately before live imaging. RPMI-free phenol red (Gibco) was used as a culture medium during live imaging.

### Fluorescence quantification

Real-time images were captured using a confocal microscope (Leica Stellaris-8) and later quantified using ImageJ. Briefly, regions of interest (ROIs) that represent areas of tubular lumen and cytoplasm, and surrounding interstitium were determined by freehand selections. Fluorescence intensities of ROIs were calculated based on a mean value of the represented wavelength on histograms in the samples of pre-treatment, 0, and 20 min of incubation. The data of 8 ROIs from at least 3 replicates were collected for analyses. Significant differences between fluorescence intensities of ROIs between d21 and d35/d49 were modeled by two-way factor student *t*-test.

### Immunostaining on whole mounted- and cryosectioned- organoid

Organoids were fixed with 4% PFA (Thermo scientific) for 1 h. For whole mounted samples, organoids were blocked using a blocking buffer supplemented with streptavidin/biotin blocking (Vector lab). Tissue was incubated with 1st and 2nd antibodies (Supplementary Table S1) overnight at 4°C and for 2 h at RT, respectively. To enhance the staining, sample clearing was performed by dehydrating the organoid samples in consecutive EtOH treatments at 50, 75, and 100%. To generate cryosections, organoids were soaked in 30% sucrose overnight, and subsequently embedded in OCT compound (Fischer Healthcare). Samples were frozen in liquid nitrogen prior to cryostat sectioning. Tissue sections were placed on glass slides and stored at −20°C. For immunostaining, tissue sections were blocked using blocking buffer supplemented with streptavidin/biotin blocking (Vector lab) and then incubated for 45 min with 1st and then 2nd antibodies (Supplementary Table S1) at RT. Samples prepared using both methods were mounted and observed under the confocal microscope.

### Bulk-RNA Seq and data analyses

Total RNA samples were collected and analyzed in the previous study (Gupta et al., 2022). Briefly, RNA samples were isolated from hPSCs (day 0), hPSC-derived NPCs (day 8), and organoids on days 21, 35, and 49 of differentiation. RNA integrity was assessed (RNA Nano 6000 Assay Kit, Bioanalyzer 2100, Agilent Technologies). Total RNA (400 ng) was used for library preparation using the NEBNext Ultra II RNA Library Prep Kit for Illumina (New England BioLabs, #E7775). Libraries were quantified using Qubit for mass concentration, LabChip for fragments distribution, and qPCR for molar concentration. The qualified RNA-seq libraries were run on NovaSeq 6000 S4 sequencers (Illumina). The MDR1 and OCT2 expression were extracted from the total bulk-RNA seq data and were also compared with developing human kidney datasets (GSE66302). Significant differences between samples were modeled by ANOVA.

## Data Availability

The datasets presented in this study can be found in online repositories. The names of the repository/repositories and accession number(s) can be found below: The RNA sequencing data used in this study is available at DDBJ Sequence Read Archive (DRA) under accession number DRA010266.

## Supplementary Material

Supplementary Video S1 Three-dimensional (3D) and Z-stack image of kidney organoid D21. **(A)** H9: 3D image (upper) and Z-stack image (bottom). **(B)** BJFF.6: 3D image (upper) and Z-stack image (bottom).

Supplementary Video S2 Real-time Rh123 uptake in d35 kidney organoid tubules after 24 min of incubation.

Supplementary Video S3 A real-time Rh123 transport in proximal tubules of organoid d30-35, after 20 min incubation **(A)** control **(B)** MDR1i **(C)** OCT2i.

Supplementary Video S4 Comparison between a real-time Rh123 transport in proximal tubules of organoid after 20 min incubation **(A)** d21 **(B)** d49.

Supplementary Figure S1 Fixation staining of organoid attached on chamber slides showed nephron tissue segmentations.

Supplementary Table S1 Antibodies used for immunostaining.
