# Supplementary material for: Live functional assays reveal longitudinal maturation of transepithelial transport in kidney organoids
Source: Front Cell Dev Biol. 2022 Aug 15;10:978888. doi: 10.3389/fcell.2022.978888 (PMC9420851; doi:10.3389/fcell.2022.978888)
Supplement: Supplementary file 2 [file Table1.docx]

**S5 table.** Antibodies used for immunostaining

|  | Gene | Manufacturer | Cat# | Dilution |
| --- | --- | --- | --- | --- |
| *Primary (1^st^) antibody* | |  |  |  |
|  | MDR1/ABCB1 | Santa Cruz | sc-13131 | 1:100 |
|  | OCT2/SLC22A2 | Abcam | ab170871 | 1:100 |
|  | PODXL | R&D | AF1658 | 1:500 |
|  | CDH1/Ecad | Abcam | ab11512 | 1:500 |
|  | bLTL | Vector Lab | B-1325 | 1:200 |
|  | PDGFR β | Abcam | ab32570 | 1:500 |
|  | CDH1 | Abcam | ab9498 | 1:500 |
| *Secondary (2^nd^) antibody* | |  |  |  |
|  | rat-Alexa 488 | Invitrogen |  | 1:500 |
|  | rabbit-Alexa 555 | |  |  |
|  | rabbit-Alexa 594 | |  |  |
|  | goat-Alexa 647 | |  |  |
|  | mouse-Alexa 647 | |  |  |
|  | strep-Alexa 750 | |  |  |
| Other markers | | |  |  |
|  | DAPI | |  | 1:1000 |
|  | Sytox Blue | |  |  |
