# Supplementary figures and images for: Live functional assays reveal longitudinal maturation of transepithelial transport in kidney organoids
Source: Front Cell Dev Biol. 2022 Aug 15;10:978888. doi: 10.3389/fcell.2022.978888 (PMC9420851; doi:10.3389/fcell.2022.978888)

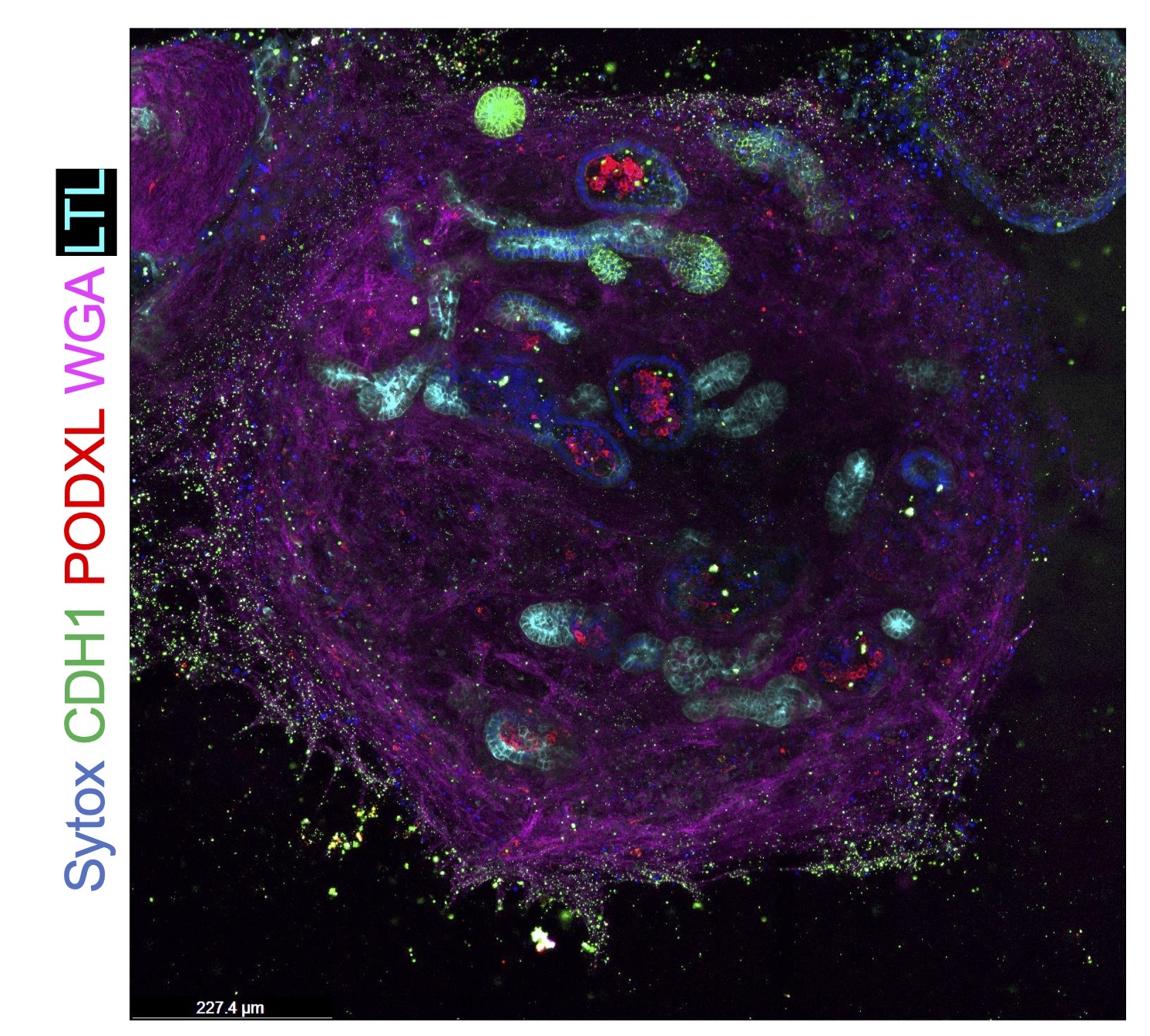

Supplement: Supplementary file 3 [file Image1.JPEG]
